# Leisure and health benefits associated with acculturation among Western international students living in South Korea

**DOI:** 10.1080/17482631.2021.1945725

**Published:** 2021-07-15

**Authors:** Junhyoung Kim, Se-Hyuk Park, May Kim, Hsueh-Wen Chow, Sua Han

**Affiliations:** a Department of Health & Wellness Design, School of Public Health, Indiana University, Bloomington, Indiana; b Department of Sports Sciences, Seoul National University of Science and Technology, Seoul, South Korea; c Department of Physical Education, Korea University, Seoul, South Korea; d Graduate Institute of Physical Education, Health and Leisure Studies, National Cheng Kung University, Tainan City, Taiwan

**Keywords:** Leisure, acculturation, Social health, mental wellbeing, international students

## Abstract

**Purpose**: Prior research provides limited information on the roles that leisure can play among Western international students studying in Eastern countries. Exploring this directional difference can provide its implications for Western international student support. Therefore, the purpose of this study is to explore leisure behaviours and leisure benefits associated with acculturation among Western international students residing in South Korea.**Method**: Using a purposeful criterion sampling strategy, a total of 18 participants (7 males and 11 females) were recruited. This study employed semi-structured in-depth interviews and a content mapping and content mining question strategy was incorporated.**Results**: The following core themes were associated with the health benefits of leisure activities experienced by international students living in South Korea: (a) promoting mental health, (b) experiencing dynamics of inter-and intra-group friendships, and (c) facilitating acculturation.**Conclusions**: The findings showed that participants developed friendships with the host individuals and other international students, became well acculturated into a new culture and experienced benefits to their mental health, indicating that leisure participation can be instrumental in improving social and psychological wellbeing.

There has been a significant growth in the number of international students across the world. When they move to a new country, these sojourners unavoidably face the challenge of navigating a new cultural and social environment. Berry et al. (2006) referred to this process of embracing new cultural values and beliefs as *acculturation*. During acculturation, international students encounter a variety of adaptation challenges associated with differences in language, culture, social norms, and educational systems (Mori, 2000; Pan et al., 2010; Sawir et al., 2008; Zhou et al., 2018). According to Yeh and Inose (2002), international students’ cultural and ethnic differences may generate intergroup anxiety and conflicts, and their struggles to adapt to their situation often adversely impact their mental health, acculturation, and academic success (Hansen et al., 2018; Taušová et al., 2018). Thus, international students have frequently reported diminished life satisfaction and quality of life (e.g., Lai, 2004; Weisman et al., 2005).

Literature on leisure studies suggests that leisure activity can serve as an effective coping strategy to deal with adaptation challenges (J. Kim, 2012). Prior studies have indicated that participating in leisure helped international students to gain cultural knowledge and effectively embrace new cultural values and perspectives (Lee et al., 2018; Zerengok et al., 2018). International students have used leisure as a way of connecting with host individuals, reducing stress, and adjusting socially, resulting in better mental health and overall quality of life. In particular, joining on-campus recreational programmes has been found to facilitate international students’ acculturation processes and their positive social interactions and relationships with others (Artinger et al., 2006; Glass, 2014; Stodolska & Alexandris, 2004).

While there is much evidence of the positive impact of leisure on acculturation and health, the majority of studies have focused on understanding leisure behaviours and leisure benefits experienced by Eastern international students who moved to Western countries (Lee et al., 2019, 2018). Little research has investigated benefits of leisure associated with the acculturation among Western international students who move to Eastern countries. While they may experience different sources of adaptation challenges from those encountered by international students in Western settings and engage in different types of leisure activities, leisure may nevertheless play an important role in facilitating their acculturation and promoting their social and psychological wellbeing. However, prior research provides limited information on the roles that leisure can play among Western international students studying in Eastern countries. By exploring this directional difference and its implications for international student support, this study expands the literature on leisure and health studies and contributes practical suggestions on ways to help international students acculturate into a new environment and promote their health and wellbeing.

For this study, we focused on Western international students studying in South Korea. According to the Korean Education Development Institute’s 2018 Analysis Report of Korean Education Statistics, the number of international students was projected to be 160,165 in 2019, and it is anticipated that the number will continue to increase dramatically every year. Despite this rapid growth, information concerning ways to support leisure and health among international students remains scant. Moreover, some studies suggest that international students studying in South Korea have experienced numerous challenges such as the language barrier, different communication styles, discrimination, homesickness, and limited social support (Ju & Kim, 2013; S. Kim, 2018; Na, 2006; Oh et al., 2015). Zhou et al. (2018), suggesting the to further examine the role of leisure engagement, which has been well documented as effective in Western settings, in helping international students’ successful adaptation and life satisfaction in an Eastern setting. Therefore, the purpose of this study is to explore leisure behaviours and leisure benefits associated with acculturation among Western international students residing in South Korea.

## Leisure behaviours of international students

Several studies have indicated that serious engagement in leisure activities can be instrumental in promoting social and psychological benefits among international students studying in the US. Artinger et al. (2006) found that recreational sports programs played an important role in socially integrating students into the university culture by encouraging bonding among participants, which resulted in strong social connections and support. In a similar line of research, Lee et al. (2018) found that serious engagement in leisure activities positively influenced school adaptation among international students attending colleges in the US. Other studies have also supported the idea that participating in leisure activities provides rich opportunities for international students to socialize with other ethnic and racial groups of students, gain cultural knowledge and understandings, and become mentally robust (Glass, 2014; J. Kim, 2012; Stodolska & Alexandris, 2004). For example, Korean graduate students who were seriously involved with sports and recreation programs were well acculturated to a new culture, coped well with academic challenges and acculturative stress, and experienced personal development (Lee et al., 2019). Also, Kanters and Forrester (2000) demonstrated that international students used leisure activities as a way of coping with acculturative stress, increasing self-esteem, expanding social networks, and enhancing life satisfaction.

On the other hand, international students often experience more barriers to participation in leisure activities than domestic students (Cho & Price, 2018). For example, Li and Stodolska (2006), who studied leisure behaviours of Chinese graduate students, found that the demands of successfully pursuing their academic goals discouraged them from participating in recreational activities, and when they did, they tended to engage in unplanned, unorganized, and passive activities, preferably with others who shared their ethnic and cultural characteristics. As these studies show, engagement in leisure activities has been shown to be critical in school adaptation and health; however previous research has limited relevance to researchers in Eastern contexts, a gap which the present study helps fill.

## Methodology

### Research design

This study employed semi-structured in-depth interviews to explore leisure benefits associated with acculturation among international students studying in South Korea. Crabtree and Miller (1999) defined the in-depth interview as “a particular field research data-gathering process designed to generate narratives that focus on fairly specific research questions.” (p. 93). Prior studies have demonstrated the effectiveness of in-depth interviews to generate narratives of lived experiences and social phenomena among people who had arduous life experiences. such as international students (Hesse-Biber & Leavy, 2005; Lee et al., 2019).

### Participants and data collection

We employed a purposeful criterion sampling strategy specifying that participants were full-time college students who were from a Western country and comfortable speaking English. We contacted the offices of international studies of various South Korean universities and obtained permission to place our flyer containing a brief description of the study on their notice boards. Potential participants contacted us through emails and phones. Before initiating the interviews, we provided participants with necessary forms and information (e.g., consent form, the study’s purpose, and withdrawal procedures). Their participation was voluntary and could be terminated upon their request. For their privacy, we used pseudonyms. The sponsoring university institutional review board approved this study.

A total of 18 participants (7 males and 11 females) were recruited. In terms of nationalities, 14 were from the USA and the rest were from Germany (1), Columbia (1), Brazil (1), and Peru (1). Their ages were between 20 and 28 years old and their lengths of stay ranged from three months to two years. Sixteen were undergraduates. The sample size was considered appropriate based on previous studies that reached theory saturation in explorations of leisure benefits associated with acculturation among immigrants and international students (Kim & Kim, 2013; Kim et al., 2012; Lee et al., 2019).

### Date collection procedure

The interviews were conducted in places determined by each participant, such as conference rooms, libraries, and coffee shops at a time of their convenience. This study used a content mapping and content mining question strategy as suggested by Legard et al. (2003). Content mapping questions were asked to derive a broad sense of each participant’s experience associated with acculturation as an international student. Examples included: “Please tell me about your life experience in South Korea.” and “What do you do when you have free time?” To capture leisure benefits associated with acculturation, mini-tour questions were used such as “Why do you participate in these activities?” “Based on your experiences, what role, if any, have these activities played in helping you deal with the challenges while you adjust to a new country?” and “Based on your experiences, what role, if any, have these activities played in helping you deal with your stress?” After each interview, each participant filled out the brief demographic survey that contained questions related to gender, education, age, country of origin, and the length of their stay. All interviews were audio-recorded and transcribed.

### Data analysis

The data were analysed based on a constant comparative method suggested by Lincoln and Guba (1985). We followed McCracken’s (1988) five steps: (a) creating raw data (interview transcripts), (b) reading each transcript for clear understanding before data analysis, (c) coding and generating basic themes from each transcript, (d) extracting major themes and illustrating them with salient excerpts, and (e) summarizing and interpreting primary and sub-themes. After creating the raw data set, each investigator read each transcript to gain a broad overview of the participant’s experience. Each investigator created a broad theme for each transcript, following which they compared their interpretations and collaboratively compared data within and among the transcripts to identify similarities and differences among the participants’ reported behaviours, experiences, and perspectives and to derive patterns across the sample, which yielded the final set of themes and sub-themes.

### Trustworthiness

Two strategies were employed to increase the credibility of the analysis. First, the research team contacted each participant to validate the themes and data interpretations. The method for respondent validation suggested by Townsend Peterson et al. (2007) was used: each participant designated our interpretations as “satisfactory” or “unsatisfactory” to express whether they were satisfied with them. Seven of the 18 participants voluntarily participated in this member-checking process. After they had reviewed our interpretations of the data, they all expressed their satisfaction. In addition, each investigator participated in the data analysis. Using constant comparative analysis, as suggested by Lincoln and Guba (1985), we collaboratively compared interviewees’ experiences, and through deliberate discussions and negotiations, we reached consensus on data generation and interpretation.

## Findings

The participants identified a variety of leisure-time activities in which they engaged such as physical activities, volunteering, outdoor recreation, travelling, and culture-related activities, from which they indicated they realized health benefits. The following core themes were associated with the health benefits of leisure activities experienced by international students living in South Korea: (a) promoting mental health, (b) experiencing dynamics of inter-and intra-group friendships, and (c) facilitating acculturation.

### Promoting mental health

Promoting mental health is the most salient theme that emerged from the interviews. All of the participants mentioned that engaging in leisure activities helped them reduce acculturative stress and increase their self-esteem and confidence. They shared that as international students they encountered a variety of adaptation challenges such as the language barrier, differences in culture and social norms, academic challenges, and homesickness. They believed that through leisure engagement, they developed the ability to cope with these challenges and reduce acculturative stress. For example, Celine (female, 23), who enjoyed socializing and exploring new environments, stated that spending time walking around the city with her friends and exploring different sites helped relieve her stress. Ginna (female, 24) stated, “Traveling is always good for your mental health or to open your mind, and meeting new people, making new friends, listening to new languages.” In a similar manner, Brittany (female, 22) stated,

In my free time I did not do much back home because it was sort of scary to go out and walk by myself … . [Here] it was nice to do something different this time, especially since I am in the huge city, and I lived in a very small town in America, so there was not a lot I can do there.

She also commented that running at night was a new activity in which she frequently engaged, and which significantly reduced her stress.

Participants also reported that leisure participation increased their self-esteem and confidence, resulting in experiencing greater happiness. For example, some were teaching English to elementary school students as volunteers. Through this activity, which involved interacting with Korean children, they experienced pleasure and enhanced self-esteem. Peter (male, 23), who practiced Taekwondo, found that demonstrating his Taekwondo skills and techniques helped him gain confidence and self-esteem. He also indicated that Taekwondo engagement facilitated his understanding of Korean culture and language, which positively and significantly affected his confidence and self-esteem.

Some participants engaged in outdoor recreation activities such as hiking, walking on the trails, and visiting parks and rivers. They mentioned that they could easily access such outdoor resources, and some joined outdoor clubs. Engaging in these activities made their lives seem happier and more productive. For example, Pedro (male, 26) stated, “I like to hike, I like to take pictures, make good memories, and I really like to walk around, to feel fully, like, how Korean life is. I am so excited about my life here.”

He also mentioned that like many people he visited parks and trails at night, where he felt safe and enjoyed his engagement.

In addition, some participants indicated that they engaged in new activities such as going to a *Noraebang* (a place with rooms equipped for singing and dancing) and a *Jimjilbang* (a place with saunas, hot and cold pools for bathing, entertainment lounges, food court, and sleeping rooms). They shared that fully immersing themselves into these activities that represented Korean culture gave them enjoyment and happiness. According to Cookie (female 21),

We don´t really have a karaoke or that kind of culture back home so it´s fun. And I always saw that in dramas, so I always wanted to try it. And the first time I went with my KUBA [Korea University Buddy Assist] group, it was so much fun. Everyone took turns singing and we had a good night. We were there forever, it was so many hours, and we didn´t notice.

She also stated that she experienced positive feelings and emotions by singing and dancing at *Noraebangs*.

Based on participants’ statements, leisure provided rich opportunities for them to experience increased happiness, self-confidence, and self-esteem and to reduce acculturative stress, which contributed to their mental health.

### Experiencing dynamics of inter-and intra-group friendships

Another benefit that leisure engagement offered was experiencing the dynamics of inter-and intra-group friendships. They mentioned that as international students, at first they had limited opportunities to form friendships with host individuals due to differences in language and cultural values and beliefs. They stressed the importance of leisure engagement as a way of breaking down these barriers and creating friendships with host individuals. They mentioned that sharing similar interests and experiences through leisure activities helped them connect with Koreans and develop intergroup friendships. According to Shellby (female, 20), who took art lessons,

From drawing I was able to make new friends and I was able to make like Korean art style, it is really minimalistic, you don’t like to use a lot of detail, but it was interesting, and I made a lot of new friends; we go shopping, we always see something weird happening, or something interesting and it was really cool to be able to hang out with my friends this last couple weeks. So that was really nice.

She valued the friendships with Koreans she made through art activities, which expanded her social network. In addition, Peter (male, 23), who as mentioned above practiced Taekwondo, interacted mainly with Koreans in this activity, with whom he developed, and he maintained friendships and who helped him with acquiring advanced skills and techniques.

Most of participants joined the language exchange programs and student activity clubs offered by their university, in which they participated in various activities and sports. For example, Shelly (female, 20) stated that she made good friends with Korean students in such clubs. Also, Ashley (female, 22) stated,

I started through KUBA and that was really helpful. They helped me a lot and from there I met some friends from by buddies. And different people around. And group projects helped a lot too, making friend in class, making groups projects and then meeting them outside of class.

She also mentioned that her involvement in student club activities allowed her to learn how to interact with Koreans in an appropriate manner, such as bowing, using two hands, and sharing food.

In a similar manner, Chris (male, 22) stated,

I just wanted to make Korean friends, cuz’ like in America, when you join clubs, you do that because you are interested or you want to use it as like a benefit to like social network, so I was like … . This is an opportunity for me to join, cuz’ they have like a club fair, and I was interested to see what they were doing, so I joined … so the main reason why I joined was to make Korean friends and be like physically active, and I think those are the two reasons.

Besides developing intergroup friendships through leisure activities, participants also interacted with other international students and fostered intragroup friendships in the context of leisure activities. They mentioned that discovering shared cultural backgrounds and ethnic identities with other international students helped them develop friendships. In addition, sharing similar experiences and challenges associated with acculturation fostered social relationships. For example, most participants created group networking for activities such as travel, shopping, sports, and outdoor programs. For example, Brittany (female, 21) stressed the importance of her friendships with other foreigners:

I do not have any problems in communicating with my travel friends as we speak English and share lots of things together as foreigners … . We travel a lot and enjoy [each other’s] company … we [have] easily bonded and supported each other.

As these examples indicate, involvement in leisure activities provided opportunities for participants to interact with and develop friendships with both host individuals and fellow international students.

### Facilitating acculturation

Participants shared that leisure helped them acquire cultural knowledge, understand different ways of communication and interactions, and behave in a more culturally sensitive manner. They expressed appreciation of engaging in outdoor programs, physical activities, volunteering, and travelling for cultural understanding and cultural knowledge. Chris (male, 22), who was passionate about soccer, joined the soccer club, where he played with Korean college students and gained cultural understandings, including culturally appropriate ways of showing respect for other players. Similarly, John (male, 23), who was a soccer team member, mentioned that he learned the importance of age in Korean culture as the leader was always the oldest in the group. He also found that socializing after each game was more important than winning or losing.

All of the participants explored historical and cultural sites to learn about the history, language, and culture of Korea when they had free time. For example, Brittany (female, 21) travelled to different cities in Korea and discovered their unique features. She stated,

When I traveled in America, each state had its own unique perspectives and culture and history. Just like each state, South Korea has rich cultural and historical features. Last week, I went to Gyeongbokgung Palace and learned about the historical relations between Korea and Japan. It was fun to understand some historical facts.

After gaining cultural knowledge, she felt more comfortable with interacting with Koreans.

Participants who volunteered as English teachers in schools also learned Korean language from students. They mentioned that the school system and the relationship between teachers and students was totally different from those in their countries. For example, Mandy (female, 22) appreciated the way her students treated her, observing that Korean elementary school students showed more respect and appreciation to teachers than students in America. Cookie (female, 21) learned some Korean language and basic communication skills from her students. She stated that she enjoyed learning a new language and communicating with others in Korean.

While the participants shared some challenges and struggles in their efforts to embrace new cultural values and beliefs, they used leisure activities as a way of being more comfortable with interacting with Koreans. For example, Peter (male, 23) explained how he learned to accept collectivistic values and beliefs:

Maybe this is something bad about me, but I feel a little intimidated if I go to a place where there is a lot of Koreans … . Maybe I have a lot of egocentrism … That will be kind of a barrier that makes me feel intimidated. I like to be in Korea, in Korean crowds, but sometimes they will be like, what is this foreigner doing here? … after I learned Taekwondo, I made lots of Korean friends and understood why Koreans liked to be collectivistic.

By providing opportunities for participants to gain cultural knowledge, promote cultural understandings, and behave in culturally appropriate ways, participation in leisure activities positively affected the students’ acculturation.

## Discussion

This qualitative study captured some of the benefits afforded by leisure activities associated with acculturation among international students from Western countries studying in South Korea. The findings showed as a result of engaging in various forms of leisure activities, participants developed friendships with the host individuals and other international students, became well acculturated into a new culture and experienced benefits to their mental health, indicating that leisure participation can be instrumental in improving social and psychological wellbeing. In particular, the results of this study stress the importance of leisure as a way of developing cultural and ethnic understandings, reducing acculturative stress, and developing valued inter-and intra-group friendships.

Prior studies have demonstrated the positive effects of leisure activities on the social integration and psychological health of international students who moved to Western cultures (e.g., Lee et al., 2019, 2018). The findings of this study indicate that Western international students in an Eastern country also experienced increased happiness, self-esteem, and confidence through leisure engagement. Despite the frequently cited differences between Eastern and Western cultures, this study demonstrates that just as leisure activity participation can positively support the acculturation of Eastern students in Western countries, the converse is also true, as Western international students were found to gain similar benefits from their participation in leisure activities in Eastern countries. Thus, this study suggests that leisure participation may be instrumental in promoting mental health among sojourners in any setting with unfamiliar cultural and social practices.

Studies exploring how participation in leisure activities was related to acculturation and health benefits among migrants to South Korea have also found that migrants embraced new forms of leisure, developed friendships, and gained cultural knowledge through such participation (Kim et al., 2018, 2016). These studies stressed the importance of leisure activities for providing opportunities for migrants to socialize with host individuals, which supports their acculturation, thereby improving their health, wellbeing, and life satisfaction. Previous studies have also focused on understanding the role of leisure in the acculturation of Eastern immigrants to Western countries (J. Kim, 2012; Li & Stodolska, 2006; Stodolska & Yi, 2003; Yu & Berryman, 1996). They indicated that leisure provided opportunities for immigrants to embrace new cultural values and beliefs, develop cultural and ethnic understandings, and assimilate to the mainstream. In support of these findings focused on Eastern migrants to both Eastern and Western countries, this study extends existing knowledge by showing that participation in leisure activities can facilitate acculturation and increase the mental health of Western international students in Eastern settings.

As a final point, studies of intragroup behaviours have indicated that people have a tendency to interact with others who have similar racial and ethnic backgrounds and form in-group biases and favouritism (e.g., Dovidio et al., 2008; Otten & Moskowitz, 2000; Stephan & Stephan, 2013). The findings of this study expand this perspective by showing that, while participating in leisure activities with others with similar backgrounds led the students to form connections and developing friendships based on common experiences, it also helped them to seek understanding of Korean culture and language and develop friendships with host individuals. This finding supports the fundamental premise of intergroup contact theory, that meaningful participation in cross-group activities can reduce people’s negative stereotypes and biases towards other racial groups (J. Kim, 2012; Matera et al., 2012; Zagefka et al., 2007), while suggesting that intragroup behaviour can support rather than hinder this outcome. That is, leisure participation can bridge intergroup gaps and boundaries in any direction when people engage in meaningful activities with anyone who is interested.

### Limitations and conclusion

This study has some limitations to be addressed in future research. First, while this qualitative study focused on leisure benefits associated with acculturation among international students, some important variables, such as the length of stay in South Korea, language abilities, and past leisure experiences, may affect participants’ leisure behaviour and associated health benefits. Future studies are needed to investigate how these variables are related to leisure and acculturation among participants. Second, this study focused on Western international students and presented findings based their leisure participation and acculturation. International students from Eastern countries may pursue different types of leisure activities and have different adaptation experiences. By exploring the similarities and differences in leisure and acculturation experiences between western and eastern international students, future studies may expand the body of knowledge concerning the effects of participating in leisure activities. Last, this study did not explore the participants’ past leisure pursuits and experiences and how they might have affected their leisure behaviours in the host country. Future researchers might include such data to investigate any changes in leisure among international students after migration.

This study suggests that leisure can be instrumental in promoting mental health, experiencing dynamics of inter- and intragroup contact, and facilitating acculturation among Western international students. The findings of this study support designing and implementing a variety of recreational programs and events would be important for health of international students and their successful adaption to a foreign setting. In particular, this study suggests that it is essential for leisure service providers to create recreational programs that emphasize multi-ethnic sports teams so that international students can engage and bond with members of the host country and other ethnic groups. In addition, international students may experience challenges to engage in leisure activities because of language barriers and cultural differences. By exploring and minimizing such barriers, researchers and programme planners can enhance opportunities for international students to fully engage in leisure activities and gain various associated health benefits.
